# Mentoring across difference and distance: building effective virtual research opportunities for underrepresented minority undergraduate students in biological sciences

**DOI:** 10.1128/mbio.01452-23

**Published:** 2023-12-12

**Authors:** Corey J. Knox, Faqryza M. Ab Latif, Natasha R. Cornejo, Michael D. L. Johnson

**Affiliations:** 1 University of Arizona, Arizona Astrobiology Center, Tucson, Arizona, USA; 2 National Summer Undergraduate Research Project, University of Arizona College of Medicine - Tucson, Tucson, Arizona, USA; 3 Department of Educational Psychology, University of Arizona College of Education, Tucson, Arizona, USA; 4 Valley Fever Center for Excellence, University of Arizona College of Medicine - Tucson, Tucson, Arizona, USA; 5 Department of Immunobiology, University of Arizona College of Medicine - Tucson, Tucson, Arizona, USA; 6 BIO5 Institute, University of Arizona College of Medicine - Tucson, Tucson, Arizon, USA; 7 Asthma and Airway Disease Research Center, University of Arizona College of Medicine - Tucson, Tucson, Arizona, USA; University of Illinois Chicago, Chicago, Illinois, USA

**Keywords:** bioinformatics, education, microbiology, virtual, research program

## Abstract

The National Summer Undergraduate Research Program (NSURP) is a mentored summer research program in biosciences for undergraduate students from underrepresented backgrounds in science, technology, engineering, and mathematics (STEM). Conducted virtually over 8 weeks every summer starting in 2020, NSURP provides accessible and flexible research experiences to meet the needs of geographically diverse and schedule-constrained students. Drawing from mentee reporting and surveys conducted within the NSURP framework involving over 350 underrepresented minority undergraduate students over three cohorts (2020–2022), matched with mentors, this paper highlights the potential benefits of students participating in virtual mentored research experiences. In addition to increased access to quality research experiences for students who face travel or academic setting constraints, we found that virtual mentoring fosters cross-cultural collaborations, generates novel research questions, and expands professional networks. Moreover, this study emphasizes the role of virtual mentorship opportunities in fostering inclusivity and support for individuals from underrepresented groups in STEM fields. By overcoming barriers to full participation in the scientific community, virtual mentorship programs can create a more equitable and inclusive environment for aspiring researchers. This research contributes to the growing body of literature on the effectiveness and the potential of virtual research programs and mentorship opportunities in broadening participation and breaking down barriers in STEM education and careers.

**IMPORTANCE:**

Summer Research Experiences for Undergraduates (REUs) are established to provide platforms for interest in scientific research and as tools for eventual matriculation to scientific graduate programs. Unfortunately, the COVID-19 pandemic forced the cancellation of in-person programs for 2020 and 2021, creating the need for alternative programming. The National Summer Undergraduate Research Project (NSURP) was created to provide a virtual option to REUs in microbiology to compensate for the pandemic-initiated loss of research opportunities. Although in-person REUs have since been restored, NSURP currently remains an option for those unable to travel to in-person programs in the first place due to familial, community, and/or monetary obligations. This study examines the effects of the program's first 3 years, documenting the students’ experiences, and suggests future directions and areas of study related to the impact of virtual research experiences on expanding and diversifying science, technology, engineering, and mathematics.

## INTRODUCTION

It has been shown that diversity increases innovation and ideas and promotes more innovative and creative solutions (1). Additionally, diverse teams are more likely to develop new and novel questions and methods as multiple perspectives and experiences lead to innovative and potentially disruptive ideas and approaches (2–4). However, despite ongoing calls to increase diversity in science, technology, engineering, and mathematics (STEM) fields in the United States, there continues to be a significant underrepresentation of Black, Indigenous, and Latino [referred to in this paper as underrepresented minority (URM)] individuals in the STEM workforce (5, 6). Although the number of URM students receiving bioscience degrees has increased, the overall percentage of URM employees in the biosciences workforce has remained stagnant (Table S1) (5). Additionally, studies have shown that despite increased enrollment in bioscience undergraduate programs, URM students are less likely to complete their degree than their non-URM peers (7). This trend highlights the need for targeted interventions and support systems to address the underrepresentation of URM, low-income, and first-generation college students in bioscience fields.

### Importance of mentored research experiences

Mentored research experiences (REs) increase students’ science aspirations and identity, increasing the likelihood of applying and enrolling in graduate programs and graduating with science degrees (3, 4). Research Experiences for Undergraduates (REUs) connect coursework and disciplinary knowledge to scientific innovation and increase engagement and retention in STEM degrees and majors. Participation in REUs improves retention rates for undergraduate degree completion, advanced degrees, and the STEM workforce (8–10). Additionally, REU participation further contributes to STEM pathway retention through strengthening students’ research abilities, sense of belonging, and supportive networks through mentoring relationships (11). A sense of belonging is especially important for first-generation, minoritized, and other underrepresented students and can be reinforced through strong mentor relationships and peer networks (12). It has been shown that URM student participation in mentored research experiences resulted in gaining skills and knowledge often associated with success in STEM fields, such as research competency and increased science identity(13). Furthermore, increased research competence later influenced students’ further involvement in research, improved academic performance, and led to a higher probability that students would remain in STEM (e.g., graduate school and research careers) (14).

However, participation in intensive mentored research opportunities can be limited due to the time and expense it could entail for undergraduate students and the availability of willing research mentors. Undergraduate students, specifically those who are first-generation and members of underrepresented groups in higher education, may have caregiving responsibilities or have financial constraints and cannot always easily take advantage of in-person research opportunities during the academic year or in the summer due to full-class schedules, family responsibilities, and work obligations (15). This highlights the need to explore additional avenues for creating and providing intensive mentored research opportunities that are accessible to students from all backgrounds. With the rise of platforms such as Zoom and other video conference software, virtual mentorship has emerged as a means of addressing accessibility barriers to research experiences.

### Mentoring from a distance: virtual research experiences

Virtual mentoring and research experiences saw a significant increase in popularity during the COVID-19 pandemic, particularly in the summer of 2020. Research indicates that virtual mentoring not only offers social, academic, and career support but also fosters the development of transferable and technical skills, akin to the advantages of in-person mentoring. Additionally, virtual mentoring offers additional benefits, including enhanced flexibility in scheduling, expanding, and strengthening national and international networks (16, 17), and the ability to conduct research and gather data from multiple locations (18).

While entirely virtual undergraduate research experiences are relatively new, there is an emerging body of research that documents their advantages (19–21). Virtual research and mentoring programs effectively address barriers to participation, such as financial constraints, transportation difficulties, living expenses, and caregiving responsibilities. The program described in this article details the utility and accessibility of using a virtual platform for an REU program to provide mentored research experiences to URM students across the United States.

### The National Summer Undergraduate Research Project

The creation of the National Summer Undergraduate Research Project (NSURP) responded to the increased need for research experiences due to COVID-19 laboratory and university closures. The program was conceived and founded by Associate Professor of Immunology Dr. Michael Johnson, himself a member of an underrepresented group in STEM and biosciences, along with significant efforts from co-founders Drs. David Baltrus and Jennifer Gardy. The foundation of NSURP and its values of accessibility, community, and diversity were inspired by Dr. Johnson’s personal experiences of challenges with support and inclusion throughout his scientific career trajectory (18). As NSURP was created to serve URM undergraduate students, NSURP speciﬁcally identified and recruited minoritized undergraduate students in microbiology and related bioscience majors. In its inaugural summer (2020), through social media and professional networks, NSURP recruited over 150 faculty, staff scientists, postdocs, and graduate trainee mentors representing 30 states, 7 (7) countries, and 95 universities, colleges, government organizations, or private labs to virtually mentor 249 undergraduate students from across the United States, all on a volunteer basis (18, 21). This effort was largely driven by the microbial science community to which Drs. Johnson, Baltrus, and Gardy belonged. Communication platforms for recruitment were limited the year NSURP began in 2020 due to the short 11-day turnaround time between program conception and implementation. However, in NSURP’s second year of 2021, funding from the National Science Foundation RAPID and REU grants not only allowed for broadened recruitment through conferences, meetings, and the newly established NSURP website but also allowed for compensation for NSURP participants for a 40-hour work week and additional staff support.

**Fig 1:**
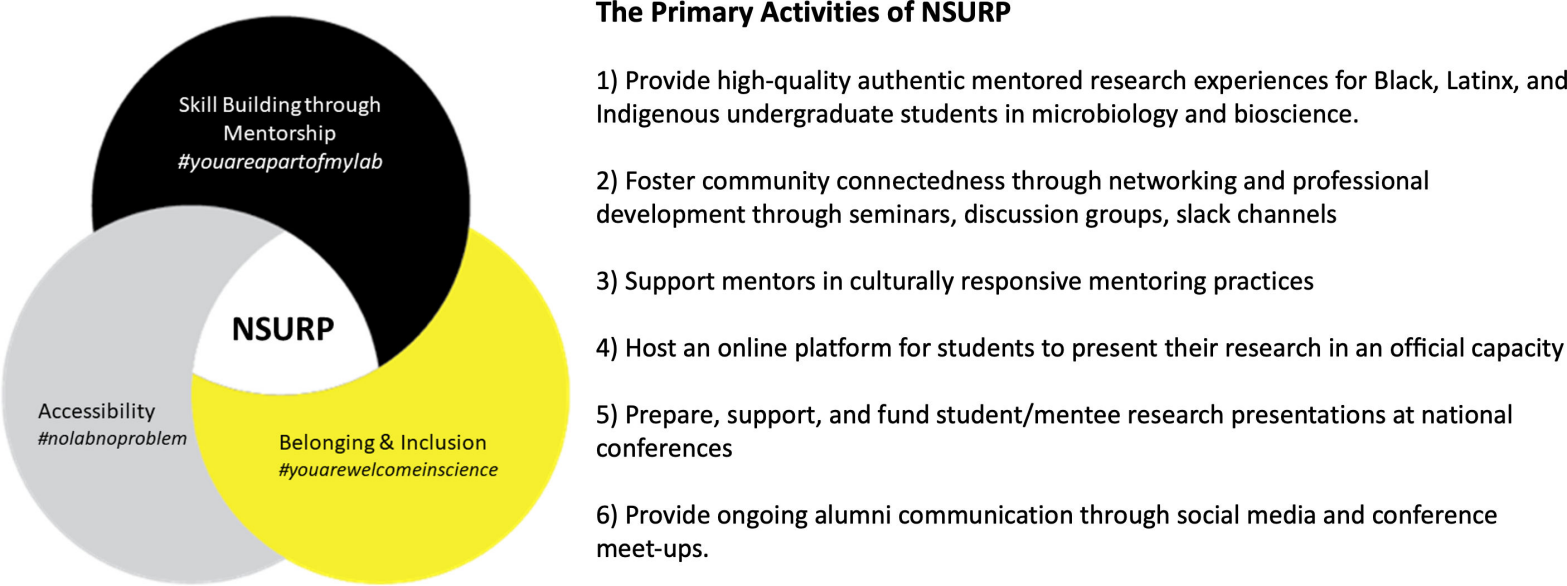
Overview of the goals of the NSURP.

Since that first summer of 2020, students (mentees) and scientist mentors have worked together on applied research projects in biosciences, with strong emphasis on microbiology, during an 8-week research internship that included professional development/graduate school preparation, guest seminars from URM professors and scientists, and virtual networking and engagement activities (Fig. 1). Research projects undertaken by mentee-mentor pairs have ranged from environmental and applied microbiology, public health microbiology, clinical and diagnostic microbiology, medical microbiology, and cancer biology (Table S2). Notably, many of these research projects contained bioinformatic or computational aspects, as these methods seemed to translate well to the virtual nature of NSURP. Throughout the years, the goals of NSURP have evolved past its original purpose of providing research opportunities for URM undergraduate students during the pandemic era. Now, NSURP seeks to address further unmet needs of URM students in science by providing meaningful virtual summer research experiences, fostering a unique scientific community between and within cohorts, and creating a foundational platform to support its participants in building their scientific careers and identity (Fig. 1).

Here, we highlight the results, advantages, and possibilities of expanding and improving research opportunities for URM undergraduate students through virtual/long-distance mentored research experiences. We present data focused on the following two research questions that address the core structure of the NSURP program:

1. Accessibility: How do mentees report the accessibility and need for virtual research opportunities?
2. Science Skill Building: How do mentees perceive the program’s impact on science confidence, science identity, and future aspirations?

We address these questions through a University of Arizona IRB-approved voluntary study using data collected during the first three NSURP cohorts in 2020, 2021, and 2022. Surveys of mentees focused on their views about the virtual aspect of the NSURP program, their acquisition of research skills, and their confidence and sense of belonging as a scientist (2108179924). By analyzing pre- and post-survey data from three cohorts, we discovered a pressing need for research opportunities that are both accessible and adaptable, allowing them to fit seamlessly with students’ locational and scheduling needs. Our data also supports that virtual programs can be used as an effective training tool and platform for summer research opportunities and can also address accessibility barriers to onsite, in-person research opportunities. Lastly, virtual research and mentoring programs have the potential added benefit of broadening science engagement and literacy outside of the scientific community, as undergraduate mentees are likely participating in the virtual opportunity while in their communities and family homes. As such, our data demonstrates that NSURP mentees were more likely to engage in scientific discussions with people in their community, such as family members and friends who, during the academic year, were not typically in the mentee’s immediate daily interactions. Taken together, our data summaries reflect that the implementation and continuation of NSURP programming continue to highlight novel emerging areas that, upon further research and investigation, can create more meaningful, effective, accessible, and culturally responsive opportunities for URM students.

## MATERIALS AND METHODS

The following data and data summaries are based on surveys from mentees who participated in the NSURP, an 8-week summer virtual mentored research program. Data is included from the 2020, 2021, and 2022 cohorts. Some questions have been changed or added over the years, which will be noted in our discussion of the data. Information on the home institution of mentees was garnered from mentee application forms. All data collection tools and procedures were IRB-approved through the University of Arizona. All incoming mentees were asked to take a pre-program survey (online) administered during week 1 of the 8-week program. The post-survey was given at the end of the last week of the program.

To assess the program’s effectiveness and its impact on the mentees, we created both pre- and post-program surveys. These surveys were distributed 1 week before program commencement and during the final week of the program. Our survey construction drew upon the program’s explicitly defined goals and current research on high-impact practices associated with the retention, progression, and sense of belonging among undergraduate students in STEM. Additionally, we incorporated questions aimed at gauging the mentees’ satisfaction levels. Furthermore, our surveys included demographic inquiries and questions concerning the mentees’ previous experiences with research activities.

We integrated items from previously validated surveys widely used in STEM education research to allow comparison and contribution to the research in STEM undergraduate education. Furthermore, the survey design was weighted to address the constructs of belonging and science identity. The items were related to academic self-efficacy in STEM, science identity, expectancy for a STEM career, and STEM belonging. The science identity and belonging items were adapted from a validated scale by reference (22).

Additionally, items were included related to confidence in specific research practices, such as data analysis, understanding the scientific literature, and generating a hypothesis which we measured both before the program and after completion. Although these items were based on self-report, confidence in science practices has been shown to correlate with persistence, higher graduation rates, increased professional benefits, and an increased interest in, and a higher likelihood of, persisting in a science degree and enrolling in graduate school (23, 24). This multi-pronged approach to survey construction was intended to align with research areas of importance, particularly for URM students in STEM, and to evaluate and assess the components of NSURP and their impact on mentees. See Table S3 for survey items.

Response rates were high across all three cohorts, ranging between 75% and 99% response rates. We have also conducted one-on-one interviews with a small sample of mentees, but those data are omitted from this study. Participants were not required to participate in either survey (pre or post).

Following participation in NSURP, students were asked to respond to 10 questions/statements related to their sense of belonging, science identity, confidence, and future aspirations through a post-program survey. These items were intended to measure the impact on the student’s sense of belonging in science, their identity as scientists, their confidence as scientists, and their intentions to pursue future opportunities in science. The virtual model of this research experience included the added community-building activities and the presence of scientists of color as guest presenters (weekly) and directors/scientists and facilitators who are members of underrepresented faculty/Ph.D. students. As entirely virtual research experiences, especially those involving full-time summer work, are rare and novel, collecting data related to skills acquired, confidence, and science identity is essential.

Depending on the data type, survey questions were analyzed using various methods. Categorical variables were analyzed using χ^2^ tests of independence or tests of proportion or were reported in frequency distributions. Items with Likert scales were analyzed by calculating the means. The 10 items on a sense of belonging, science identity, confidence, and future aspirations were measured on a scale of 1–5, with 1 = *strongly disagree* and 5 = *strongly agree*. The percentages of mentees who responded *agree* or *strongly agree* with the items and the mean score of each item are shown in Table 3.

## RESULTS

### NSURP demographics

In the past 3 years (2020–2022), the program has had 364 URM undergraduate mentees. Students were from 127 educational institutions, including Historically Black Colleges and Universities, Hispanic-Serving Institutions, and community colleges (Table 1). Notably, 55% of the participants were first-generation college students, 68% identified as female, and 48% of the 2021 and 2022 cohorts (we don’t have this data for 2020), identified as low income/Pell grant eligible (Table 1). In 2021, we paired 65 mentees representing 44 universities with mentors and in 2022 (year 3), 54 mentees representing 29 universities and 3 community colleges. The slight decline in participants was due to available funding for the students. This was not due to demand, as the applications received in 2021 and 2022 were both over 300. Notably, the large difference between the numbers of the 2020 cohort vs the cohorts in 2021 and beyond is due to the following reasons: (i) this was the height of the pandemic for individuals needing opportunities and (ii) all students were volunteers in 2020 and not paid as they were in subsequent years, thus decreasing the ability to serve a population of almost 250 participants.

**TABLE 1:** NSURP mentee demographics from 2020, 2021, and 2022 cohorts (*n* = 364)

	Pell eligible^*a*^	First generation	Female identifying	Racial/ethnic identity					
				Latinx/Hispanic	Black	Native American	Pacific Islander and Hawaiian	Multi-racial	Other
Total (n)	56	200	248	146	164	9	3	23	19
Total (%)	48%^*a*^	55%	68%	40%	45%	2.5%	0.8%	6.3%	5.2%

^*a*^ No data collected for 2020, data consists of 2021 and 2022 cohorts (*n = 117*).

Having same-identity mentors and role models has been shown to positively affect persistence and belonging in STEM among UR undergraduates (25, 26). However, due to the low number of URM individuals in biosciences and STEM, it is less likely that URM students will have access to similar or same-identity role models in their fields. This representation was mirrored in NSURP, where at least 85% of mentors identified as white. Still, there was significant geographic diversity in that mentors have represented 113 institutions, including universities, government research institutions, and several private research labs worldwide.

### Accessibility: broadening participation in research programs

In our first NSURP launch during the COVID-19 pandemic, we asked participants if they could participate in a summer research experience if there were no shutdowns due to the COVID-19 pandemic. We were surprised that 35% of the students did not expect they would be able to participate in an onsite experience, and 25% were unsure (Table 2). A test of proportion yielded a result of *z* = 1.091, *P* = 0.862. These results were echoed by the 2021 cohort even when many onsite programs were restarted. In 2021, we asked the cohort if they would have been able to participate in an onsite internship, and 20% of the students responded in the affirmative, 48% were unsure, and 32% answered that they would not have been able to do so (Table 2). A test of proportion gave a result of *z* = 0.321, *P* = 0.625.

**TABLE 2:** Accessibility: challenges to in-person REUs

	If it weren't for COVID shutdowns, would you have been able to participate in an onsite research internship?	Would you have been able to participate in an onsite research internship?
	2020 (n= 100)	2021 (n= 54)
No	35%	32%
Unsure	25%	48%
Yes	40%	20%

In an open-ended question, the most often cited reasons for choosing a virtual/remote internship were family responsibilities, part-time jobs, financial difficulty (specifically housing costs and transportation), and flexibility with schedule, with two students mentioning disabilities that would make it more challenging to be in an onsite lab setting. We have no further data on why students chose “not sure” other than they were likely variations of the student comments that said no. In the 2022 application cycle, we included and weighted selections based on a question asking the applicant why a virtual REU was necessary. As such, we did not include the 2022 cohort in this analysis, as our selection criteria skewed the results. Notably, the stipends for these students starting in 2022 also included a budget for food and housing, as those budget items are included in the in-person programs. We speculate that this additional student budget, however, does not fully offset all the caregiving, health, or financial constraints and responsibilities.

**Fig 2:**
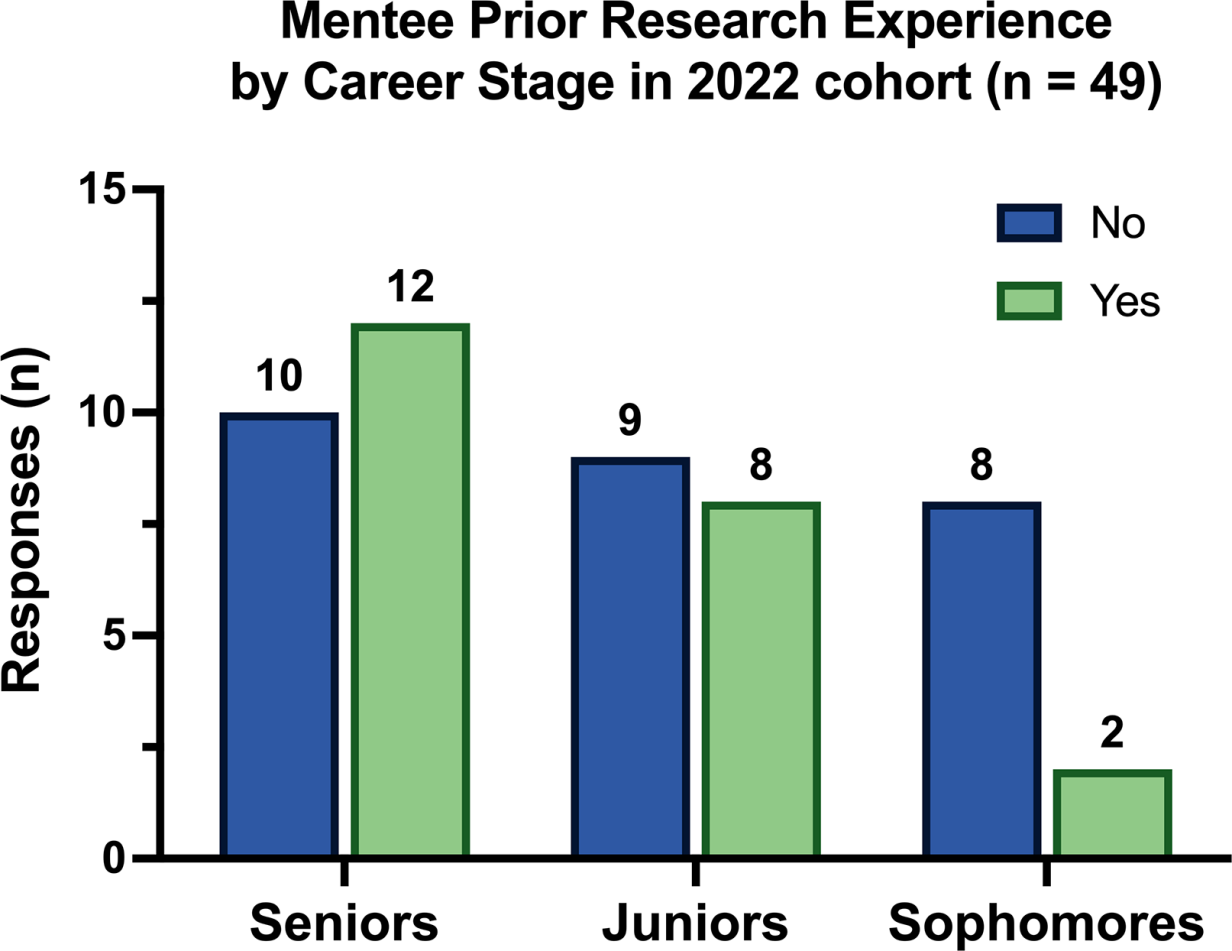
Mentee research experience prior to NSURP for the 2022 cohort, categorized by career stage (*n* = 49).

We also asked participants about their previous research experiences. In our questions, we differentiated between mentored REUs and being a member of a laboratory team or coursework that included labs. While we were not surprised by the low frequency of REUs of first and second-year students who are all STEM-related majors (most in the Biosciences), we were surprised by the number of juniors and seniors in the 2022 cohort for whom NSURP would be their first mentored research experience (Fig. 2). Of the 50 mentees from the 2022 cohort who responded to the question (*n* = 50), 38% identified as juniors and seniors with no research experience before NSURP. A χ^2^ test of independence yielded a result of X^2^ (2, *n* = 49) = 3.365, *P* = 0.186.

**Fig 3:**
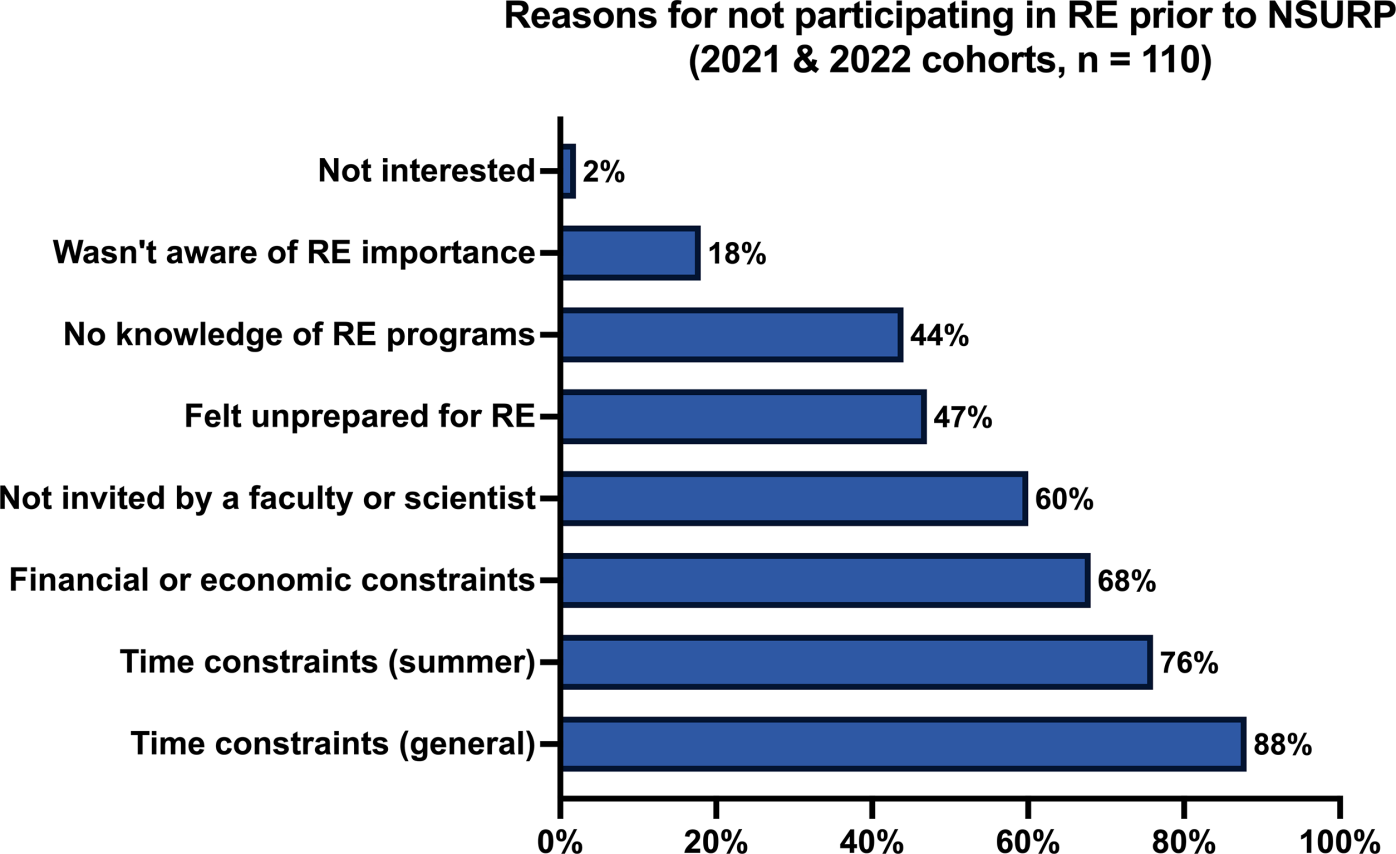
Mentee reasons for not participating in research experience prior to NSURP (*n* = 110). Responses of “strongly agree” and “agree” are represented in these data.

We next asked a multiple-choice question asking why students had not participated in a research experience in the past (2021 and 2022 cohorts). The most frequent reason was time constraints, followed by financial and economic constraints (Fig. 3). Not as highly rated as a barrier were reasons connected to how research experience programs are advertised, how these opportunities are shared by faculty, and student perception about who they are for—i.e., more academically prepared students. These are all reasons related to accessibility and how these opportunities are communicated to students.

### Science skill building: how do mentees perceive the program’s impact on science confidence, science identity, and future aspirations?

There are a variety of skills that are positive indicators for scientific outcomes such as seeing oneself as a scientist in the moment and seeing oneself as a scientist moving forward (27). Based on the mentees’ responses, NSURP positively impacts science confidence, preparedness, sense of belonging, and identity (Table 3). The results are based on the responses of the 2021 and 2022 cohorts, with 54 students responding to the items in the 2021 cohort (*n* = 54) and 40 students responding in 2022 (*n* = 40). For most items, over 80.00% of the mentees agreed or strongly agreed, with mean scores averaging above 4.30. However, for the item, *I feel more confident about preparing an application to a graduate research-based or medical school program*, only 75% of the mentees agree or strongly agree, with a mean score of 4.03, in the 2022 cohort. Lastly, we also note in Table 4 the high satisfaction of almost all mentees on core program outcomes.

**TABLE 3:** Sense of science, belonging, identity, and confidence (2021; *n* = 54 and 2022; *n* = 40 cohorts)

Item	2021 Cohort		2022 Cohort	
	Agree or strongly agree	*M*	Agree or strongly agree	*M*
My abilities as a scientist grew.	98.15%	4.69	92.50%	4.50
I am better at asking scientific questions.	90.74%	4.50	90.00%	4.30
I am more confident as a scientist.	90.74%	4.57	87.50%	4.32
I am more likely to pursue a career in STEM.	88.89%	4.59	87.50%	4.47
My scientific communication skills have improved.	92.60%	4.65	92.50%	4.40
I am more likely to apply to a research-based or medical school program.	87.60%	4.44	87.50%	4.38
I feel more confident about preparing an application to a graduate research-based or medical school program.	85.60%	4.43	75.00%	4.03
I feel more confident regarding taking future science courses.	92.60%	4.44	95.00%	4.42
I feel more confident in pursuing future scientific research opportunities.	96.30%	4.61	95.00%	4.58
I feel that I belong and can succeed in science.	87.03%	4.56	87.50%	4.38

**TABLE 4:** Mentee ratings of NSURP program outcomes (2021 and 2022) *n* = 104

Selected mentee ratings of the program overall	Agree/strongly agree
I felt supported by my mentor.	94%
My abilities as a scientist grew.	96%
I am more confident as a scientist.	90%
I am more likely to pursue a career in STEM.	91%

## DISCUSSION

The NSURP program continues to evolve as we gain a better understanding of the imperative of providing flexible and diverse virtual programs. These programs aim to foster the growth and diversification of the community of future scientists in the biosciences and across STEM fields. While the immediate catalyst for creating virtual research programs was the COVID-19 pandemic, our data clearly shows that even as labs and research sites have reopened, many bioscience students are still not benefiting from mentored research experiences during their undergraduate journey. For instance, our findings indicate that 38% of juniors and seniors in the 2022 cohort had no prior research experience before participating in NSURP (see Fig. 2).

As we continue to work with mentees and mentors in various modalities—virtual, hybrid, and in person, it is essential to continue to examine not only who is being reached but also how these programs are recruiting, preparing, and supporting mentors to provide culturally and personally relevant research experiences, establishing virtual and sustainable networks for students/mentees, and measuring the long-term outcomes and retention of mentees, especially those who are underrepresented in STEM. The data highlighted in this paper focused on three crucial components and outcomes that we believe are vital to evolving more flexible and accessible high-impact training experiences for undergraduate students.

### Virtual mentorship opportunities add accessibility that can help diversify STEM

Virtual mentorship opportunities in STEM can help diversify the field by providing access to mentoring and support for individuals from underrepresented groups who may face financial barriers to participation in traditional in-person mentoring programs and barriers related to caretaking, family responsibilities, transportation, and scheduling conflicts (16, 17, 21). For example, our findings show that some students of color struggle to allow time to move or live away from home for summer in-person programs due to economic/financial barriers, caretaking, and other reasons (Table 2). These reasons were indeed prominent in the reasoning provided by the 2022 applicants on why they desired a virtual research opportunity. With an increased implementation of virtual mentored research programs, STEM fields can be diversified. Based on past literature (16, 17, 21, 28–30), virtual REUs support the diversification of STEM fields through:

1. Access: Virtual mentorship can provide access to mentors and resources for individuals who live in remote areas or lack the financial means to travel to in-person mentorship programs.
2. Flexibility: Virtual mentorship can offer more flexibility in scheduling and communication, which can be especially beneficial for individuals with busy schedules or caretaking responsibilities.
3. Inclusivity: Virtual mentorship can create more inclusive and safer spaces for individuals facing social or cultural barriers to participating in traditional in-person mentorship programs.
4. Exposure: Virtual mentorship can expose individuals to diverse perspectives, experiences, and resources from mentors and peers worldwide, which can broaden their understanding of STEM and its applications.

The virtual aspect of NSURP, along with other virtual lab and mentorship programs, not only broadens accessibility and reach but also enriches the depth and relevance of research projects. These projects can now be more closely intertwined with students’ communities and family networks. Furthermore, virtual programs enable national and global engagement, facilitating expanded networking opportunities.

Mentees expressed a sense of connection to students from across the nation, highlighting this as a positive and enriching outcome of the program. One distinctive feature of NSURP, compared to traditional REU programs, is that our mentors are spread worldwide, albeit primarily within the US. In fact, faculty members had their own dedicated community channel on our Slack page. Consequently, this diversity of perspectives contrasts with those from a single institution or region. This diversity offered improved access for exchanging information, graduate school advice, and establishing connections. It also exposed participants to new research domains and opportunities.

### Cross-identity virtual STEM mentoring

During each year of the program, we have increased the resources through measurement and programming to build culturally responsive mentoring and programming through guest speakers, discussion topics, small-group virtual meetings, and research and evaluation that explored these topics. As mentioned previously, across the 3 years of the program, 100% of mentees were UR students, while across the 3 years, 82% of mentors identified as white/European-American. This is not unique to NSURP but reflects the demographics of the microbiology/biology fields and a trend that NSURP is working to disrupt. At US universities, URM researchers make up only 3.5% of the faculty in the life sciences and 6.3% of the faculty in basic science departments at medical schools (31). Evidence shows that same-race/ethnicity and gender mentoring relationships can positively affect the success and retention of underrepresented students in STEM fields by increasing students’ persistence and perceived level of support (32–35). Additional studies suggest that same-race/ethnicity and gender mentoring can lead to an increased sense of belonging, reduced isolation, and improved academic and career outcomes for students of color in STEM (36). URM students bring unique perspectives to research from their lived experiences and are more likely to know firsthand about specific health needs, challenges, and contexts of their respective communities, thus supporting the likelihood that research questions, strategies, and methods are culturally appropriate and meaningful. Given that NSURP recruits students from UR populations, NSURP mentors should be willing and prepared to engage in discussions, instruction, and opportunities that support the identities and aspirations of mentees. Fuller and Torres Rivera (37) applied a culturally responsive approach to center students’ lived experience and cultural knowledge at the forefront, increasing student engagement, success, and retention in a microbiology laboratory course. This study found that students were more engaged, better understood the course content, could communicate scientific information better, and had increased retention when a culturally responsive approach was used.

Culturally responsive mentoring can validate the contributions of racially minoritized students’ cultural histories, identities, experiences, and worldviews, and it encourages mentors to evaluate their prejudices, biases, and attitudes (38). The capacity for mentoring across differences using culturally responsive frameworks is vital for URM students’ success in higher education and broadening participation and leadership in higher education and STEM. Mentors who have completed some form of training reported higher levels of efficacy in the mentoring domain, which includes skills related to managing their biases or prejudices that could potentially affect their mentoring relationship (39). They also reported higher communication, expectation management, and professional development skills (40). To support underrepresented students in STEM fields, we strongly suggest that mentor preparation programs should include cultural awareness training to enhance mentors’ knowledge and attitudes toward structural or societal barriers that may hinder their mentees’ persistence in STEM (41). Mentees from underrepresented populations may also benefit from discussing issues related to their identity, culture, or other identities with their mentors, highlighting the significance of cultural competence in mentor preparation (42).

Summer programs that depend on unpaid volunteer mentors may struggle with the expectation of requiring additional preparation or training of mentors. We fully acknowledge that the NSURP mentors are volunteers who are not compensated for their summer work with mentees and other program activities and recognize that this fact further highlights their allyship. Summer programs have a short window of interaction with mentors before the beginning of the program. The NSURP program provides orientation, resources, and other tools for mentors. NSURP continues to build and explore resources and opportunities for mentors to engage with mentoring resources and external programs that can enhance cross-cultural mentoring skills without placing more unpaid work expectations on mentors. As more universities support and reward their faculty for training and applied experience in culturally responsive teaching and mentoring, NSURP, in a real world and not class-based manner, can support these skill-building opportunities for faculty and scientists.

### Science skills building and science identity and belonging

The final topic of the findings described above relates to the efficacy and effectiveness of virtual mentoring programs to positively impact science skill building, confidence, and identity. Our mentee feedback reflected positive outcomes. Over the years, there has been the assumption that virtual modalities are not as effective as in-person lab experiences in skill building and creating relationships and feelings of belonging. However, feedback from our mentees over the past 3 years tells us that students, even those who have participated in in-person REUs, view this virtual mentorship as adding to their science and research knowledge and skills and positively influencing their science identity and sense of belonging in STEM (Table 3). NSURP students left the program with tangible skills such as coding, learning new software programs, and having experience with various data analyses. Increased sense of belonging and science identity have been associated with higher levels of motivation, engagement, and achievement in STEM disciplines, which then increases the likelihood of persistence, success, and well-being (43, 44).

NSURP mentors bring their personalities, expectations, and structures for communication and instruction to their mentoring relationships. This flexibility between mentees and mentors is a strength of the program format. NSURP supports mentors with resources related to time management and virtual tools. Discussion and sharing of best practices for managing a virtual mentorship are discussed at length in the orientation for mentors. As such, with proper support, we believe that virtual mentorship opportunities can create a more inclusive and supportive environment for individuals from underrepresented groups in STEM and help break down some barriers that can prevent them from participating fully in the field. NSURP has the following programming to cultivate a sense of belonging and support, which are aligned with findings from the literature regarding virtual mentored research programs (28, 30, 45). The programs below also reflect NSURP’s mission of fostering community connectedness and inclusion.

1. Virtual networking events: Organize virtual events where mentees can connect with mentors and professionals from diverse backgrounds. This can include virtual conferences, webinars, and networking sessions focusing on specific STEM fields or topics of interest.
2. Online discussion forums: Create online forums where mentees can ask questions, share ideas, and discuss challenges they may face in their STEM studies or careers. This can include general forums or forums specific to certain STEM fields or career stages.
3. Peer-to-peer engagement opportunities: Encourage mentees with similar cultural and ethnic backgrounds to mentor each other and share their knowledge and experiences. This can be especially beneficial for individuals from similar backgrounds who may share common experiences or challenges.

### Benefits of REUs “at home”: creating academic and research connections to students are culture and community

An area of continued research related to science skills and identities is to document how students working in their own communities, and sometimes family homes, which is often the case during a virtual research experience, can allow opportunities for community and culturally connected science identity, agency, and motivation. In a limited number of interviews with past mentees, we noticed that mentees expressed various unintended benefits to bringing their academic identities “back home.” Examples include increased science communication to different audiences. For example, mentees explain their science work to family members, friends, and others who would usually not be a part of the daily academic life of students. Another example is that mentees are more likely to make or see connections between their bioscience research to issues, conditions, or topics relevant to their families or communities.

Connecting science to a student’s community, culture, and everyday life can lead to many benefits, including strengthening science identity and expanding knowledge and skills through the deliberate connection between culture, activity, and concept (46, 47). Some of the ideas for extending and making these connections more intentional include the following (48–50):

1. Assign students to investigate ways or applications of their research area in their community or culture. This could be exploring health and environmental issues broadly or finding a non-profit research project or medical facility that connects their research to their own town.
2. Practice explaining their research to a friend, family member, or colleague from their community. This may also be done through narration and/or storytelling.
3. Develop a research question that addresses an issue in one’s community, neighborhood, family, or culture.
4. Create data collection procedures that can be done in students’ location/geography.
5. Provide mentees with opportunities to take mentors and fellow mentees on a virtual tour of their community or a place of significance.

### Future directions

NSURP is a dynamic program that adapts to the evolving needs of its students. For example, in 2022, NSURP introduced an additional feature called Peer Orchestrated Development (PODs) to foster a stronger sense of belonging among mentees and establish a robust and sustainable network of scientific peers. These PODs are organized and led by NSURP staff, with weekly meetings involving 10–14 mentees. Since NSURP mentees come from diverse institutions, PODs offer them a valuable opportunity to connect and build relationships with their peers. Beyond enhancing these connections, PODs also serve as a platform for scholars to engage with NSURP staff throughout the program. In 2023, PODs were composed of 8–9 scholars and featured a more structured meeting curriculum. While these changes may complicate cohort comparisons, the data we collect reflect the program’s ongoing evolution. We are currently conducting an extensive study to assess the impact of implementing PODs, and the results will provide valuable insights into their effectiveness.

While it may be challenging to generalize findings across various research experiences and virtual programs, some outcomes can still offer valuable points of comparison. Specifically, the data collected from the NSURP program can serve as a foundational resource for developing new virtual mentoring programs and provide valuable insights for individual mentors. These findings can serve as essential data sets for comparing research programs of different modalities, including hybrid and in-person approaches. The data presented in this study underscore the effectiveness of virtual mentored research opportunities, such as NSURP, as demonstrated in Table S2, which highlights a broad range of microbiology-focused research within NSURP. Future research endeavors will assess the applicability of virtual mentored research models in various research fields, disciplines, and methodologies. We speculate that virtual research opportunities such as NSURP will serve as a compliment to traditional in-person REU set-up. As the availability of in-person REUs is limited, we view virtual opportunities as an addition to the already existing REU structure to give more undergraduate students research opportunities and do not foresee virtual REU opportunities taking away from in-person REU experiences.

Also, efforts to incorporate culturally responsive practices into mentor and mentee training are under exploration. We are also interested in understanding how graduate school admission committees view virtual research experiences in comparison to in-person research experiences. However, past studies have shown that students who receive research training are more likely to apply to graduate or medical school due to their increased sense of belonging, preparedness, and confidence (51).

Lastly, NSURP will continue to strengthen its NSURP alumni community and to conduct robust follow-up studies of mentees beyond their undergraduate degrees. More broadly, we will continue to participate in the important and much-needed scholarship on how NSURP and other mentoring programs can contribute to increasing diversity and inclusiveness in STEM fields.

## SUPPLEMENTAL MATERIAL

Supplemental Tables mbio.01452-23-s0001.docx Tables S1-S3.

ASM does not own the copyrights to Supplemental Material that may be linked to, or accessed through, an article. The authors have granted ASM a non-exclusive, world-wide license to publish the Supplemental Material files. Please contact the corresponding author directly for reuse.
