# Supplementary material for: Mentoring across difference and distance: building effective virtual research opportunities for underrepresented minority undergraduate students in biological sciences
Source: mBio. 2023 Dec 12;15(1):e01452-23. doi: 10.1128/mbio.01452-23 (PMC10790749; doi:10.1128/mbio.01452-23)
Supplement: Supplemental Tables — Tables S1-S3. [file mbio.01452-23-s0001.docx]

**Mentoring across difference and distance: Building effective virtual research opportunities for underrepresented minority undergraduate students in biological sciences**

**Supplemental Information**

Corey J. Knox^1,3^, Faqryza M. Ab Latif^2^, Natasha R. Cornejo^3,4^, Michael D. L. Johnson*^3-7^

^1^University of Arizona

Arizona Astrobiology Center

Tucson, AZ, 85721

^2^University of Arizona College of Education

Department of Educational Psychology

Tucson, AZ, 85721

^3^National Summer Undergraduate Research Project

University of Arizona College of Medicine - Tucson

Tucson, AZ, 85724

^4^Department of Immunobiology

University of Arizona College of Medicine - Tucson

Tucson, AZ, 85724

^5^Valley Fever Center for Excellence

University of Arizona College of Medicine - Tucson

Tucson, AZ, 85724

^6^BIO5 Institute

University of Arizona College of Medicine - Tucson

Tucson, AZ, 85724

^7^Asthma and Airway Disease Research Center

University of Arizona College of Medicine - Tucson

Tucson, AZ, 85724

*Email: mdljohnson@email.arizona.edu

| **Supplemental Table 1** |  |  |  |
| --- | --- | --- | --- |
| *US biological sciences trainees and faculty by race and ethnicity (percentages)* | |  |  |
| **Ethnicity/Race** | **Graduate students** | **Postdoc appointees** | **Tenured Faculty** |
|  |  |  |  |
| **White, non-Hispanic** | 51.4% | 38% | 78% |
| **Black/African American** | 5.9% | 3.2% | 3% |
| **Hispanic/Latinx** | 8.5% | 5.7% | 4% |
| **Native American** | 0.3% | 1.1%** | 0.1% |
| **Asian & Asian American** | 23.8% | 44% | 13% |
| **Native Hawaiian or Pacific Islander** | 0.2% | .02% | 0% |
| **Two or more and unknown** | 10% | 8% | 1% |
| **International Students** | 18.6%* | 53%* |  |
| **Total #** | **83,213** | **21,455** | **34,900*** |
| *(NSF, 2019; NCSES, 2019)* |  |  |  |
| **The figures for international students are counted twice in both race/ethnicity and category and international.* | | | |
| *** Includes all tenured faculty. No information was available through NSF databases on the international status of faculty* | | | |

| **Supplemental Table 2** | |  |
| --- | --- | --- |
| *Disciplines and room winners for 2021 and 2022 cohorts* | | |
| **Cohort** | **Room Discipline** | **Title of Winning Presentation** |
| **2021** | Applied and Environmental Microbiology | Investigation of *Fusarium Oxysporum* |
|  | Applied and Environmental Microbiology | Analyzing Host-Microbiome Interactions in *Drosophila melanogaster* with Lactobacillus planatarum through Genome Analysis |
|  | Applied and Environmental Microbiology | Siderophore production in the bacterium *Penicillium raistrickii* |
|  | Computational Microbiology and Immunology | Identifying Pathways Involved in Colon Inflammation Induced by Cancer Immunotherapy by scRNAseq Analysis |
|  | Medical Microbiology | Application of the Competitive Lottery-Based Theory to the Cystic Fibrosis Microbiome |
|  | Clinical and Diagnostic Microbiology | The Bacteria-lorette: Exploring the Relationships between *Streptococcus Pneumoniae* with Corynebacteria and Respiratory Syncytial Virus |
| **2022** | Applied and Environmental Microbiology | Changes in Gene Expression of Group B Streptococcus in Response to Glutathione Availability and Hydrogen Peroxide |
|  | Applied and Environmental Microbiology | Bison dental calculus metabolome of ancient samples |
|  | Immunology | Investigation and characterization of *Bombella Apis* isolate DLM19 |
|  | Clinical and Diagnostic Microbiology | Clinical Metagenomics, Antibiotic Resistance Surveillance and Data Discordance: How I fell in love with the commensal microbiome and omic data |
|  | Medical Microbiology | An exploration of the patient-specific taxonomic differences in the cystic fibrosis lung microbiota |
|  | Public Health | Circulating EBV MicroRNAs in COVID Patients |

**Supplementary Table 3**

*Pre-program survey questions*

| Please read the following statements and think about yourself and rate how true the following statements reflect your own beliefs. (Likert scale from 1 to 5) |
| --- |
| I feel confident in my ability to learn the material in courses |
| I am capable of learning the material in my courses |
| I am able to achieve my goals in my courses. |
| I feel able to meet the challenge of performing well in my courses. |
| I am likely to apply to a STEM related research or medical school program in the future |
| I feel confident in my ability to learn the material in courses |
| I am capable of learning the material in my courses |
| I am able to achieve my goals in my courses. |
| I feel able to meet the challenge of performing well in my courses |
| I am likely to apply to a STEM related research or medical school program in the future |
| In general, my interest in science is an important part of my self-image |
| My interest in science is an important reflection of who I am. |
| I feel like I belong in the field of science. |
| I have a strong sense of belonging to the community of scientists. |
| I am a scientist. |
| I would enjoy a career in a science related career. |
| I would like to have a career in science. |
| I consider myself a member of the STEM community. |

| Think about a project that you have been involved in or may get involved in and indicate the extent to which you feel confident that you could complete the following tasks. (Likert scale from 1 to 5) |
| --- |
| Generate a research question to answer |
| Use scientific literature to guide research |
| Create explanations for the results of the study |
| Use scientific language and terminology |
| Conduct basic lab or virtual data analysis |
| Communicate research results to others |

| How important are the following experiences and/or goals to you through the NSURP experience? (Likert scale from 1 to 5) |
| --- |
| Network with other students/mentees |
| Network with other faculty and scientists including my mentor |
| Learning about different research areas in the Biosciences |
| Improve research skills |
| Improve coding/computer skills |
| Improve presentation and poster skills |
| Improve literature review and writing skills |
| Understand how different types of research applies to community issues |
| Learn about the work and experiences of other scientists |
| Discuss and learn about issues and advancement strategies specific to brown and black students in science and academia |
| Learn about the process for apply to graduate programs |

**Supplementary Table 3**

*Post-program survey questions*

| Please select the option that best describes your level of agreement with the following statements related to the impact of your participation in the NSURP Program. (Likert scale from 1 to 5) |
| --- |
| My abilities as a scientist grew. |
| I am better at asking scientific questions. |
| I am more confident as a scientist. |
| I am more likely to pursue a career in STEM. |
| My scientific communication skills have improved. |
| I am more likely to apply to a research-based or medical school program. |
| I feel more confident about preparing an application to a graduate research-based or medical school program. |
| I feel more confident regarding taking future scientific courses. |
| I feel more confident in pursuing future scientific research opportunities. |
| I feel that I belong and can succeed in science. |

| Please select the option that best describes your level of agreement with the following statements related your experience in the NSURP program. (Likert scale from 1 to 5) |
| --- |
| I felt supported by my NSURP mentor. |
| I received advice in addition to things that were project specific. |
| I received advice beyond project specific guidance. |
| I plan to ask my NSURP mentor for a letter of recommendation. |
| I would recommend NSURP to other undergraduates. |
| I would participate in NSURP again. |
| I attended or listened to more than half of the BIPOC seminars. |
| I found the BIPOC seminar series helpful. |
| I plan to use my NSURP project to present at local, regional, or national meetings. |
| I found NSURP enjoyable. |
| I learned something new. |
| I will actively seek out other research experiences in the future |
| My research project or ongoing summer research is an issue/topic relevant to my lived experience or community of origin. |
| I was satisfied with my final research presentation |
| I was well supported in preparing my final presentation |

| Please select the statement that reflects your own reasons for not previously (prior to NSURP) participating in mentor-led research projects/experiences. |
| --- |
| I didn't know about opportunities for research |
| I had time constraints during the school year due to course load |
| I had time constraints during the summer due to family or other employment |
| I have/had financially or economic factors |
| I haven't been invited to work with faculty or other scientists |
| I wasn't aware it was important |
| I was not interested in this a participating in a research experience |
| I didn't feel I was prepared |

| Please indicate how frequently each of the following occurred in your relationship with your primary research mentor. (Frequency scale, ranging from ‘never’ to ‘always’) |
| --- |
| My mentor created opportunities for me to bring up issues of race/ethnicity as they arose. |
| My mentor encouraged me to think about how the research related to my own lived experience. |
| My mentor was willing to discuss race and ethnicity, even if it may have been uncomfortable for them. |
| My mentor raised the topic of race/ethnicity in our research mentoring relationship when it was relevant. |
| My mentor approached the topic of race/ethnicity with me in a respectful manner. |
| My research project or ongoing research question could connect to an issue/topic relevant to my lived experience or community of origin. |
